# Synergistic Effect by Polyethylene Glycol as Interfacial Modifier in Silane-Modified Silica-Reinforced Composites

**DOI:** 10.3390/polym13050788

**Published:** 2021-03-04

**Authors:** Minghan Xu, Hao Xue, Wit Yee Tin, He Wang, Zhanfu Yong, Qingfu Wang

**Affiliations:** Key Laboratory of Rubber-Plastics, Ministry of Education/Shandong Provincial Key Laboratory of Rubber-Plastics, School of Polymer Science and Engineering, Qingdao University of Science & Technology, Qingdao 266042, China; minghan.xu@qust.edu.cn (M.X.); xuehao85362@hotmail.com (H.X.); w.wityeetin@gmail.com (W.Y.T.); wh2009aa@163.com (H.W.); 03496@qust.edu.cn (Z.Y.)

**Keywords:** interfacial modifier, polyethylene glycol, silica, magic triangle properties, green tire technology

## Abstract

The viscoelastic behavior and reinforcement mechanism of polyethylene glycol (PEG) as an interfacial modifier in green tire tread composites were investigated in this study. The results show a clear positive effect on overall performance, and it significantly improved all the parameters of the “magic triangle” properties, the abrasion resistance, wet grip and ice traction, as well as the tire rolling resistance, simultaneously. For the preparation of the compounds, two mixing steps were used, as PEG 4000 was added on the second stage in order to avoid the competing reaction between silica/PEG and silanization. Fourier transform infrared spectroscopy (FTIR) confirmed that PEG could cover the silanol groups on the silica surface, resulting in the shortening of cure times and facilitating an increase of productivity. At low content of PEG, the strength was enhanced by the improvement of silica dispersion and the slippage of PEG chains, which are chemically and physically adsorbed on silica surface, but the use of excess PEG uncombined with silica in the compound, i.e., 5 phr, increases the possibility to shield the disulfide bonds of bis(3-(triethoxysilyl)-propyl) tetrasulfide (TESPT), and, thus, the properties were deteriorated. A constrained polymer model was proposed to explain the constrained chains of PEG in the silica-loaded composites on the basis of these results. An optimum PEG content is necessary for moderately strong matrix–filler interaction and, hence, for the enhancement in the mechanical properties.

## 1. Introduction

Today, silica-based green tire treads have a great importance, as they are superior to carbon black-loaded tire treads regarding the “magic triangle” properties: rolling resistance, wet skid performance and abrasion resistance [1,2,3]. The “magic properties” depend on each other in a complicated manner, because it is difficult to improve one of these properties without the deterioration of one or two of the others, due to the interdependence of them. Therefore, it is one of the most important abilities of the green tire technology to improve the three magic properties simultaneously.

The surface of precipitated silica is covered with a polar and hydrophilic layer of acidic silanol groups. The dispersion of silica in nonpolar rubbers such as styrene–butadiene copolymers (SBR), polybutadiene (BR), etc., is significantly more difficult to achieve than for carbon black [4]. As a consequence, the silica surface has to be silanized in order to make it hydrophobic [5,6]. The sulfur containing silane coupling agents, such as bis(3-(triethoxysilyl)-propyl) tetrasulfide (TESPT) and bis (3-(triethoxysilyl)-propyl) disulfide (TESPD), are commonly used, which have a potential to create sulfur linkages between silica surface and the unsaturated polymer matrix and greatly improve the polymer–filler interaction [7,8,9,10,11,12,13,14]. Compared to the TESPT, TESPD has a higher temperature stability and, therefore, improved scorch during silanization when high mixing temperatures are required. The silica–silane system provides greater safety on wet roads and makes tires more energy efficient, but it adversely affects the abrasion resistance in comparison to tires filled with carbon black. The usage of silane coupling agent only for silica modification is hardly enabled an improvement in all three magic triangle parameters simultaneously [15].

In our previous research, the immersion of silica-loaded vulcanizates in deionized water results in the permeation of water into the vulcanizates. The absorbed water has a huge effect on the dynamic mechanical properties of silica-loaded green tire tread vulcanizates. A new tan δ peak around −5–0 °C was formed after swelling in water, and the height of this peak increased with the amount of absorbed water. The interaction between silanol groups and water molecules occurs by hydrogen bonds [16], resulting an increase of loss factor (tan δ) at 0 °C, which demonstrates the improvement of wet grip performance and reductions of loss modulus (G”) and tan δ at 60 °C, which are indicators for lower rolling resistance of a tire. Water was preferentially adsorbed around the silica surface rather than in the polymer matrix. Hence, the silica aggregation, as well as the filler–filler interaction, can be reduced by the physisorption of water. However, these beneficial influences can be removed by drying when the physisorbed water is desorbed [17]. 

Therefore, the present study emphasizes the use of a kind of interfacial modifier instead of water molecules, which can physically adsorb or/and chemisorb on the silica surface and will not show the reversibility in the vulcanizates. The selected interfacial modifier should have a phase transition temperature, which is a main factor to influence the dynamic mechanical performance and other physical properties in polymer science, closed to the phase transition temperature of water (or to the best in the range of −30 °C to 0 °C), because the higher tan δ at −20 °C and 0 °C are indicators for the better performances of ice traction and wet grip. The interfacial modifiers, such as polyethylene glycol, cetyltrimethylammonium bromide, polyoxyethylene sorbitan, ionic liquids and different amines are commonly used in silica modification [18,19,20,21,22,23,24,25,26]. Polyethylene glycol (PEG) was chosen here due to a series of molecule weight that can be found, and the different molecule weights normally results in the difference of glass transition temperature. As a result, PEG 4000 with the Tg of −22.4 °C [27] was used in the compound study. Furthermore, rarely have publications reported the filler–rubber interface and mechanism of PEG in silica-reinforced green tire tread composites. The silane coupling agent of TESPD was selected to be used together in silica modification. The silica-filled rubber master batches were prepared in three mixing steps, and PEG 4000 was added after silanization in the second mixing step in order to avoid the competitive reaction between silica/silane and silica/PEG. In this part, the properties of the composites containing different contents of PEG were investigated. Moreover, in order to better understand the hydrophilic nature of silica, silica was firstly modified by TESPD in solution, and, after the silanization reaction, a different amount of PEG was added to the system. The liquid PEG 400 was used in the solution study. The modified silica was characterized by Fourier transform infrared spectroscopy (FTIR) and thermal gravimetric analyzer (TGA) in order to understand the interaction mechanisms of silica and PEG by the results of these characterizations.

## 2. Materials and Methods

### 2.1. Materials

A solution-polymerized styrene butadiene rubber (S-SBR) grade RC2557-S and a solution high-cis polybutadiene polymer (BR) grade BR9000, both from Sinopec Beijing Yanshan Petrochemical Co., Ltd. (Lanzhou, China), were used for the preparation of the compounds. The S-SBR has a vinyl content of 53 wt.% and a styrene content of 23 wt.% and is extended with aromatic oil (24.3 wt.%). The filler used was precipitated silica (Ultrasil^®^ VN3) with a specific surface area (CTAB) of 160 m^2^/g from Evonik Degussa (China) Co., Ltd. (Beijing, China). The silane coupling agent, bis(3-(triethoxysilyl)-propyl)disulfide (TESPD), was obtained from Evonik Degussa (China) Co., Ltd. (Beijing, China). The antioxidant Vulkanox^®^ 4020NA and the ozone wax Antilux^®^ 500 were supplied by Lanxess (China) Co., Ltd. (Shanghai, China) and Rhein Chemie (Qingdao) Co., Ltd. (Qingdao, China). Two kinds of PEG (molecular weight: 400 and 4000 g/mol) were obtained from Xiya Reagent (Shandong) Co. Ltd. (Linyi, China). All of the ingredients were industrial grades and used as received.

### 2.2. Silica Modification

The reaction model was established in the solution state. We added 1 g silica and 125 mL deionized water to each flask. The TESPD solution with a known concentration (80 ppm) was obtained by diluting appropriate standard stock solutions with methanol. We pipetted 1 mL of the standard solution containing 0.08 mg TESPD into each flask. All silica slurries were heated to a temperature of 80 °C under high-speed stirring for 1 h. Then, different content of PEG 400, with a concentration of 62.5 ppm, was added into the flasks, respectively, according to the formulation as shown in Table 1. The slurry was stirred for another 1 h, and the modified silica slurry was then obtained. The modified silica powders were extracted in a Soxhlet extractor using ethanol for 8 h to remove the residual TESPD and PEG. Then, the extracted silica powders were dried in an oven at 120 °C for 24 h and prepared for characterization.

### 2.3. Compound Preparation and Vulcanization

Table 2 shows the formulations of the compounds studied in this paper. Three mixing steps were utilized to prepare the compounds. In the first mixing step, the rubbers and silica were mixed in an internal mixer. Then, silane (TESPD) was added, and the mixing temperature was raised to 150–160 °C and held for 3 min. After dumping the compounds, they were stored at ambient temperature overnight. Subsequently, a second mixing step mix was used, and PEG 4000 was added, during which the temperature was again raised to 150–160 °C for 3 min. Finally, the vulcanizing package was added on a mill at approximately 50 °C in the third mixing step. The vulcanization was performed at 160 °C for the specific t90 of each compound, which was previously monitored with a GOTECH Rheometer.

### 2.4. Characterizations

#### 2.4.1. ATR–FTIR Spectroscopy

Attenuated total reflectance-Fourier transform infrared (ATR–FTIR) spectra were measured by a VERTEX-70 FTIR spectrometer (Bruker Co., Ettlingen, Germany) equipped with a horizontal 45° germanium (Ge) crystal ATR device at room temperature. The spectra were the results of 30 scans in the spectral range of 4000~550 cm^−1^. All of the curves have been normalized.

#### 2.4.2. Thermal Gravimetric Analyzer (TGA)

TGA were done by TG209F1 (NETZSCH, Gerätebau, Germany) at a heating rate of 10 °C/min from 30 to 800 °C in nitrogen atmosphere.

#### 2.4.3. Mooney viscosity

Mooney viscosity, ML (1 + 4) 100 °C, was performed with an Alpha Technologies MV 2000E (Anderson, SC, USA), according to Deutsche Industrie Norm (DIN) 53523.

#### 2.4.4. Payne Effect of the Composites

Payne effect of the composites was characterized on a dynamic mechanical analyzer (Model DMA/SDTA 1+, METTLER TOLEDO, Columbus, OH, USA) in the stain mode. The strain sweeps were measured from 0.45 to 13.0% at a constant frequency of 1 Hz and temperature of 25 °C. The sample dimension was 6 × 6 × 2 mm^3^.

#### 2.4.5. Tensile and Tear Properties

Stress/strain tests were performed at ambient temperature with a Zwick tensile tester (Model Z005, ZwickRoell GmbH & Co. KG, Ulm, Germany) at a crosshead speed of 500 mm/min using optical strain control (DIN 53504). For tear strength, the vulcanizates were tested at room temperature using angle-shaped rubber specimens with the same machine and speed as applied for tensile tests, according to DIN 53507. The measurements were done 5 times, and mean values were used.

#### 2.4.6. Rebound Resilience

For the measurements of rebound, the specimens with cylindrical shapes were used (diameter: 60 mm; thickness: 6 mm) at 23 °C, according to DIN 53512, with the Zwick rebound tester 5109.01.

#### 2.4.7. Compression Set

The compression set was done at 23 °C and 70 °C for 48 h, according to DIN 53517. Five specimens were tested for each formulation, and midvalues were used.

#### 2.4.8. DIN Abrasion

DIN abrasion was measured with Abrasion tester of Frank 11565, according to DIN 53516. The specimen was cylindrical shapes with a diameter of 16 ± 2 mm and a minimum thickness of 6 mm. The abrading distance on the surface of the specimens and the speed were 40 m and 40 rpm, respectively.

#### 2.4.9. Dynamic Mechanical Analysis on Vulcanizates

Dynamic mechanical analysis was characterized on a dynamic mechanical analyzer (Model DMA Q800 TA Instruments, New Castle, DE, USA) in the tension mode. The temperature sweeps were measured from −80 to 100 °C at a constant frequency of 10 Hz, strain amplitude of 0.5% and a heating rate of 3 °C/min. The sample dimension was 6 × 4 × 2 mm^3^.

#### 2.4.10. Equilibrium Swelling Experiments

By means of equilibrium swelling the crosslink density of the vulcanizates and the polymer–filler interaction was determined. The samples were immersed in toluene in closed bottles at ambient temperature on a shaker. Three aliquots of each sample were taken out periodically and removed of the excess liquid on the specimen surface by a filter paper and then weighed immediately with an electronic balance. The weight of the swollen samples was continued until reached the equilibrium. The crosslink density was determined by means of Flory–Rehner equation [28]:

$$v=\frac{-\left[ln\left(1-V_{r}\right)+V_{r}+\chi V_{r}^{2}\right]}{V_{0}\left(V_{r}^{1/3}-0.5V_{r}\right)}$$

where *v* is the crosslink density, $V_{0}$ is the molar volume of the solvent, χ is the polymer solvent interaction parameter, and $V_{r}\;$ is the volume fraction of swollen polymer, which can be calculated from the following equation:

$$V_{r}=\frac{1}{1+Q\left(\frac{\rho_{p}}{\rho_{q}}\right)}$$

in which $\rho_{p}\;$ is the density of polymer, $\rho_{q}$ is the density of the solvent, Q is the equilibrium swelling value, which is given by the equation:

$$Q=\frac{m_{2}-m_{1}}{m_{2}}$$

where $m_{1}$ is the weight of swollen polymer and $m_{2}$ is the weight of polymer before swelling.

The values of the Flory–Huggins interaction parameter χ for cured rubber with different solvents can be found in the chemistry handbooks. For toluene χ = 0.37 was used in the calculations.

#### 2.4.11. Morphological Analysis

A JEM-2100F scanning electron microscope (JEOL, Co. Ltd., Tokyo, Japan) was used to characterize the fracture surface of the vulcanizates after tensile testing. The specimens were examined after sputter coating with a thin film of gold. An accelerating voltage of 1.0 KV and a magnification range from 500× to 1000× were used.

## 3. Results and Discussion

### 3.1. Characterization of Silica Modified by TESPD and PEG

**Figure 1:**
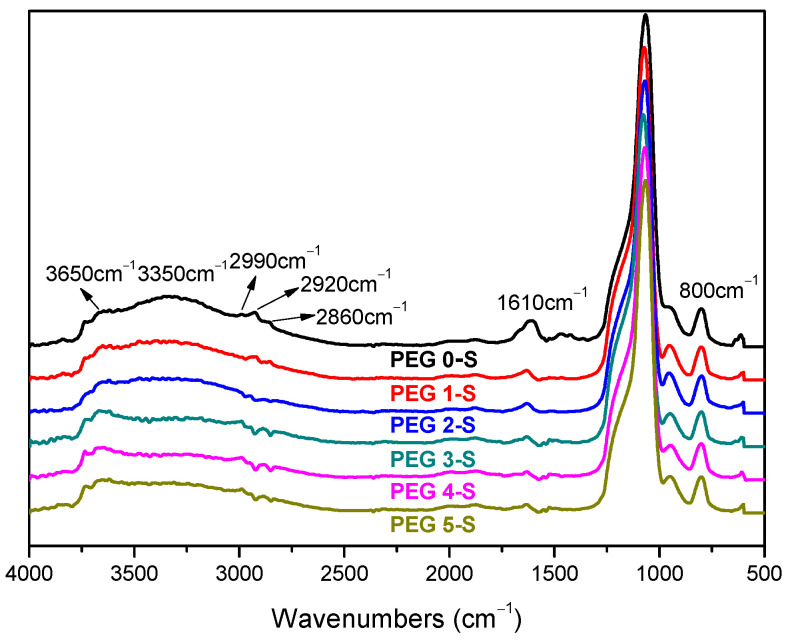
Fourier transform infrared spectroscopy (FTIR) spectroscopy of modified silica.

The FTIR spectra of the modified silica are presented in Figure 1. The absorption between 3650 and 3000 cm^−1^ is due to –OH bond stretching vibrations, and the whole band structure changes in a non-monotonous way. In the spectra, the absorption at around 3650 cm^−1^ indicated the uncombined –OH in the system; for example, the residual of free water in the mix remains almost constant intensity. However, the intensity at 3350 cm^−1^ is result from the number of the associated hydroxyls, including the silanol hydroxyls and the chemically combined water absorbed on the silica surface. Although the residual water is one of the factors influencing the intensity of these peaks, a slight increase of the water content can be found from the weight loss from 30 to 110 °C of TGA results (Table 3), which is probably due to the hydrophilic nature of PEG. The number of the associated –OH bonds is correlated to the relative intensity of the peaks at 3350 cm^−1^, when a high absorption means a large number of hydroxyl groups presented on the silica surface. The relative intensity can be calculated by using the normalized FTIR data, and the results are listed in Table 4. The relative intensity at 3350 cm^−1^ shows an abrupt decrease when PEG is added in the range of 1–4 phr and thereafter slightly rises when the PEG content is 5 phr, which means –OH groups are effectively shielded by the presence of 4 phr PEG. Furthermore, the absorption at 1630 cm^−1^ is assigned to the deforming vibrations of –OH bonds [29,30]. A sharp decrease of the peak intensity at 1630 cm^−1^ can be also observed after the modification of PEG, and the peak moves leftward, demonstrating the hydrogen-bonds generated in the systems. This also demonstrates that the amount of active –OH bonds on the silica surface are effectively shielded by an appropriate amount of PEG. The variation of the peak shape at 2990, 2920 and 2860 cm^−1^, which correspond to the asymmetrical stretching vibration and the symmetrical stretching vibration of methylene group [31,32,33], shows another confirmation of the appearance of different amounts of organic groups on the modified silica surface.

The weight loss of the modified silica in the temperature range of 110–800 °C was attributed to the dihydroxylation as well as the degradation of silane and PEG. After the addition of PEG, the modified silica expressed larger weight loss than the PEG-free sample, which clearly indicates the adsorption of PEG on the silica surface. The TGA curves of PEG 4-S and PEG 5-S were almost overlapped together, and the weight losses in the second region no longer increased. This reveals that PEG will hardly further cover the silica surface with the concentration more than 4 phr. The TGA curves can be found in the supporting information (Figure S1).

**Figure 2:**
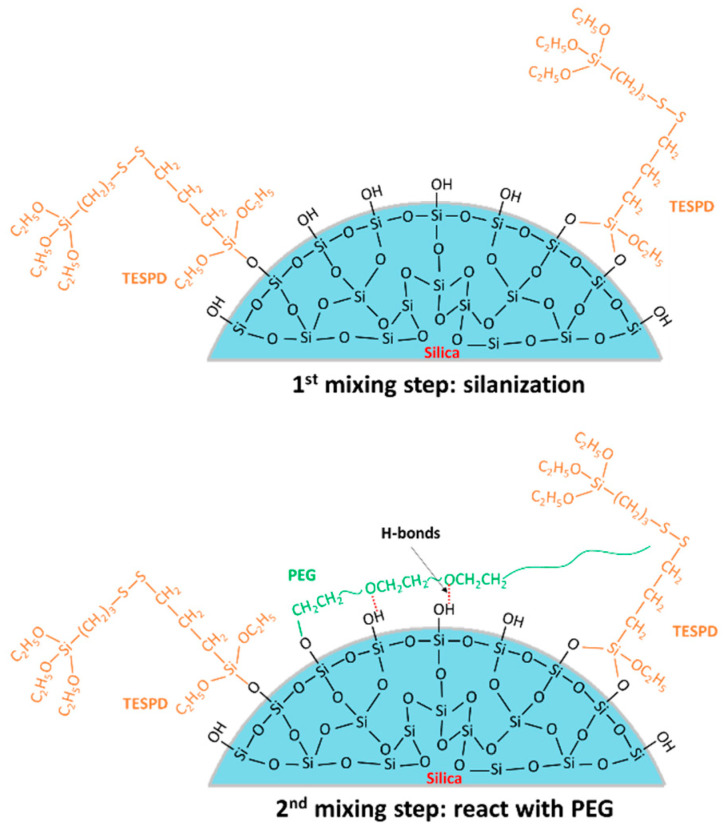
Schematic of the possible reaction mechanisms during the two mixing steps.

Based on the FTIR and TGA results, the possible reaction mechanisms of TESPD and PEG on silica surface during the 1st and the 2nd mixing steps are shown in Figure 2. On the 1st mixing step, high temperature was applied to trigger the silanization, and no PEG was added into the compounds during this stage; as a result, there is no competing reaction to affect the interaction between silica and silane. On the 2nd mixing stage, PEG was achieved to cover and shield the residual silanol groups on silica surface. Therefore, the reactivity of silica modified with TESPD and PEG together differs from that of silica modified with TESPD, alone.

### 3.2. Properties of the Unvulcanized Compounds

**Figure 3:**
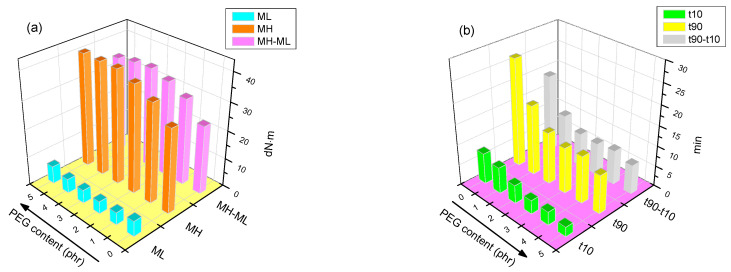
Vulcanization characteristics: (**a**) the time at 10% of curing degree (t10), optimum cure time (t90) and t90–t10 and (**b**) minimum torque (ML), maximum torque (MH) and MH–ML.

The cure characteristics were measured by the increase of the torque values as a function of time at 160 °C, according to a standard procedure with a moving die rheometer (MDR). Figure 3a,b show the vulcanization characteristics of the six composites. The value of minimum torque (ML) gives an indication for the processing properties and dispersion of fillers in the composites. As can be seen in Figure 3a, the silanized silica (PEG 0) had a greater value of ML due to the less filler networking after the silanization reaction and the large silica agglomerates. After the addition of PEG in the second mixing step, the minimum torque value was reduced significantly, caused by the improvement of silica dispersion. However, after being organically modified by 5 phr PEG, the ML is larger than PEG 0 composite, which is probably due to the enhanced viscosity by the excess amount of PEG in the composites. The maximum torque (MH) values of the composites with PEG are quite higher than that of the PEG 0 composite without PEG, owing to the increase of the polymer–filler interaction. The delta torque values (MH–ML) give an indication for the overall crosslink density [34,35], including the polymer–polymer, the polymer–silane–silica and the filler–filler interactions. The difference between MH and ML for the PEG composites are larger than PEG 0 composite, indicating that the interactions increase in the presence of interfacial modifier PEG in the composites. The crosslink density shows the highest value when 3 phr PEG was added, which is probably due to a synergistic effect by PEG and silane coupling agent in the filler–polymer network of the composites: PEG promotes the silica dispersion, which resulted in the increase of the silanization efficiency. The excess content of PEG not only acts as a lubricant but also increases the possibility to cover and shield the disulfide bonds of the TESPD and results in the reduction of crosslink density.

**Figure 4:**
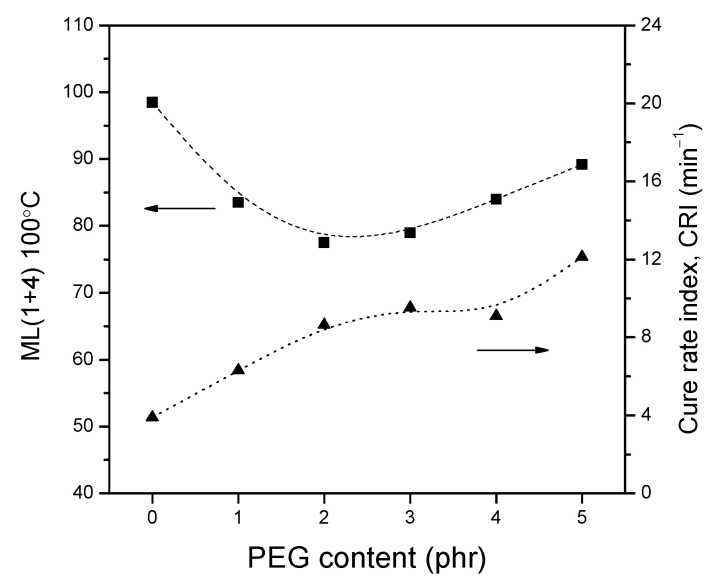
Mooney viscosity and cure rate index of the composites with different polyethylene glycol (PEG) contents.

The values of the scorch time (t10) and the optimum curing time (t90) can be used to evaluate the vulcanization rate. As illustrated in Figure 3b, with the addition of PEG in the composites, the vulcanization time gradually decreases. The highly polar silanol groups on the silica surface are known to have negative effects on the vulcanization, because of the acidic nature of silica and the accelerator adsorption [7]. Despite the modification of silica by a silane coupling agent, there are only approximately 15–35% of the silanol groups on the silica surface which can react with silanes, due to the steric effects of the ethoxy groups of silanes and the limited accessibility of the silanol groups [36]. As a result, the vulcanization efficiency is significantly improved, caused by shielding the residual acidic polar hydroxyl groups on silica surface by PEG and by reducing the retardation effects of vulcanization. The difference between the t90 and t10 tended to decline after the surface modification of silica by both silane and PEG, which indicates that the vulcanization rate tended to increase. The only silane-modified silica compound (PEG 0) showed much longer scorch and cure times with lower cure rate index (Figure 4). Therefore, there should be fewer free silanol groups left in the PEG modified composites.

The addition of PEG into the silane-modified silica composites also affected the Mooney viscosity (Figure 4), which is in accordance with the values of minimum cure torques. The Mooney viscosity decreased sharply when PEG is added in the range of 1–2 phr and, thereafter, slightly rose when the PEG content exceeded 3 phr. This phenomenon may be related to filler dispersion and networking. The addition of a small amount of PEG led to an improvement of silica dispersion and polymer–filler interaction. Once the PEG amount was high enough in the compound to act as lubricant and reduce the shearing force, the viscosity increased, due to the high filler–filler interaction.

**Figure 5:**
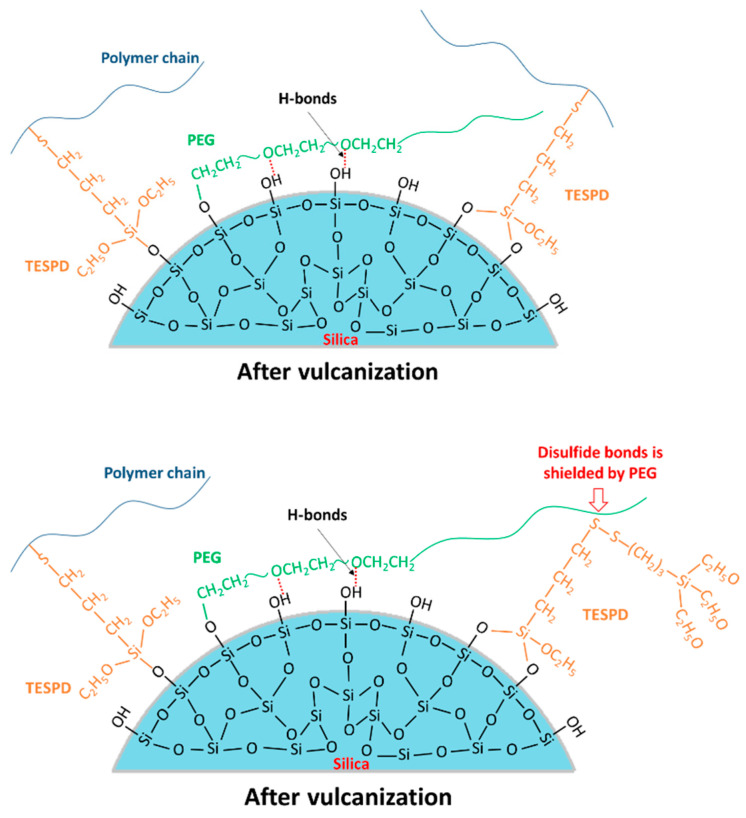
Schematic of the possible reaction mechanisms after vulcanization.

Based on the results of the cure characteristics, the possible reaction mechanisms of TESPD and PEG on silica surface after vulcanization are shown in Figure 5. It is clear that PEG could cover and decrease the silanol groups on silica surface, reduce the polarity natural of silica, as well as improve the dispersion of the polar silica in nonpolar rubber matrix. However, when high-content PEG was added in the system, it enhanced the possibility to shield the disulfide bonds of the silane and resulted in the reduction of crosslink networks during vulcanization. Very likely, chemical and hydrogen bonds between PEG and unsilanized silanol groups presented in silica play a decisive role in the dispersion of silica agglomerates.

### 3.3. Payne Effect of the Composites

**Figure 6:**
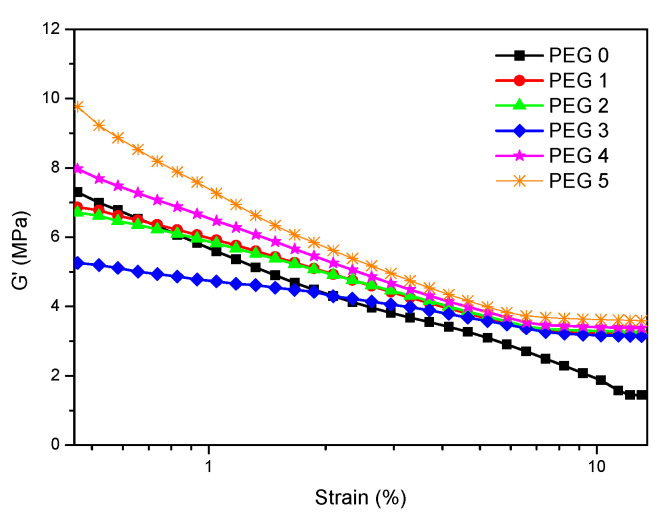
Dependencies of storage moduli G’ on the strain amplitude (Payne effect).

In order to characterize filler aggregation in the composites, DMA strain sweeps were performed at 25 °C, and the dependency of storage moduli (G’) on the strain amplitude was determined by the difference between G’ at low and high strains (Payne effect). A larger difference, i.e., higher Payne effect, means a higher degree of filler–filler interactions, as well as a poorer filler dispersion. The results in Figure 6 indicate that the Payne effect significantly reduces in the presence of small amount of PEG. At a low amount of PEG below approximately 3 phr, the Payne effect ΔG’ tended to decrease from 5.86 MPa (PEG 0) to 2.11 MPa (PEG 3), but there was a further rise at higher amounts, indicating an increase in filler network formation. The use of PEG at 5 phr increased the Payne effect again, similar to that of the reference PEG-free compound. The small amount PEG decreased the filler–filler interaction and, thus, played a positive role in the silica dispersion. More contents of PEG acting as lubricant could reduce the viscosity during mixing and generate less shearing forces to break down the silica agglomerates/aggregates. So, the final composites show high viscosity (Figure 4) as well as high filler–filler interaction.

### 3.4. SEM Images of Fracture Surface

**Figure 7:**
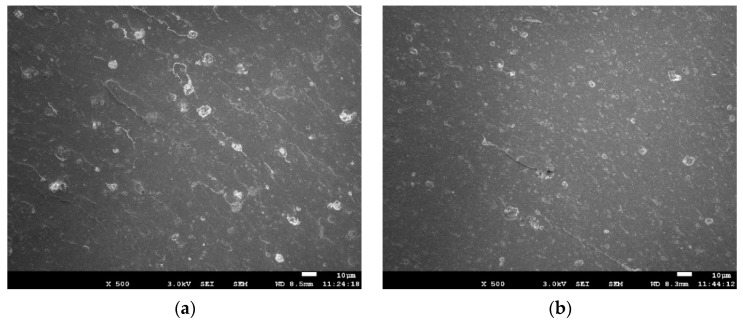

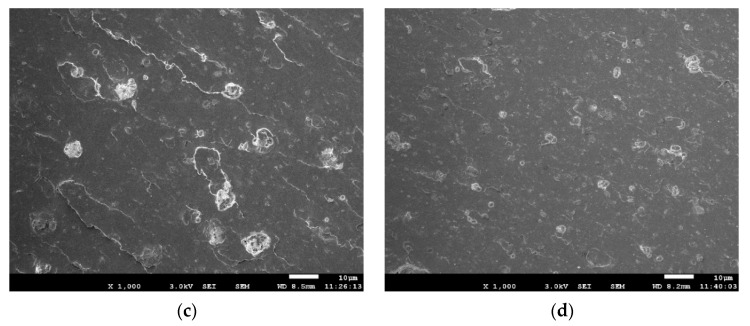
Scanning electron micrographs of PEG0 and PEG4 composites with two magnifications. (**a**) PEG0 with ×500 magnification; (**b**) PEG4 with ×500 magnification; (**c**) PEG0 with ×1000 magnification; (**d**) PEG4 with ×1000 magnification.

The effect of PEG on the dispersion of silica was studied by SEM on the fracture surfaces of tensile testing, which were taken from PEG0 and PEG4. The respective micrographs are given in two magnifications (Figure 7). Figure 7a,c give the micrographs which were obtained from the PEG-free composite. Comparing with the PEG0 composite, Figure 7b,d show micrographs (in two magnifications) from PEG4. The results show that the silica agglomerates are more visible and reveal that, without the addition of PEG, silica dispersion is much poorer. The more qualitative results gained by SEM are in good agreement with the observations from the Payne effects as well with the results from the physical mechanical properties.

### 3.5. Equilibrium Swelling Experiments

**Figure 8:**
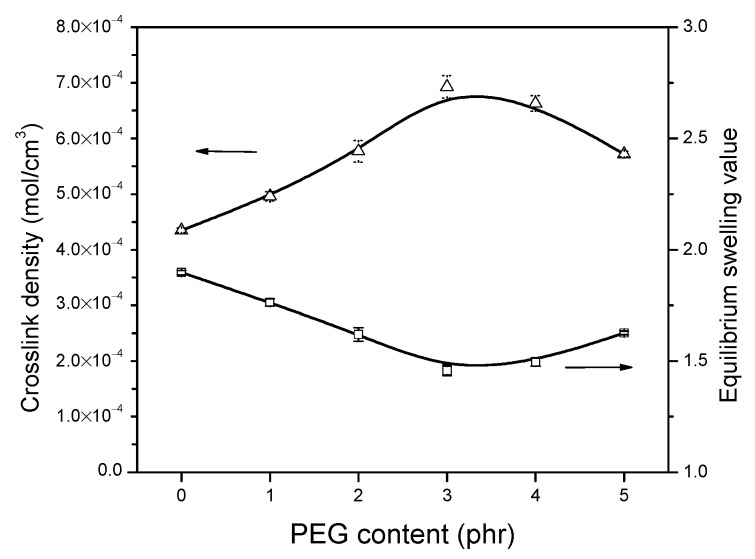
Crosslink density and equilibrium swelling value of the composites with different PEG contents.

To determine the effect of the filler–rubber interaction due to silica, silane and PEG on the crosslink density in the vulcanizate structure, the rubber contents were each divided into the silica contents to calculate the values per 1 phr of filler contributing to the crosslink density. As can be seen in Figure 8, the addition of PEG has a great effect on the crosslink density, which shows higher values than the composite without PEG (PEG 0). The crosslink density of the compounds displayed an optimum point, i.e., the highest filler–rubber interaction, and also at 3 phr of PEG addition in the composite, the same tendency as observed for delta torque values (MH–ML) of MDR. A further increase of PEG content reduced the crosslink density in agreement with the possible reaction mechanisms (Figure 5), as described earlier. The increase in filler–elastomer interaction when a small amount of PEG was added resulted in a restriction in filler mobility and, therefore, less flocculation. However, once the concentration of PEG was high, on the one hand, it acted as lubricant, and on the other hand, disulfide bonds could possibly be shielded by PEG.

### 3.6. Physical Mechanical Properties

**Figure 9:**
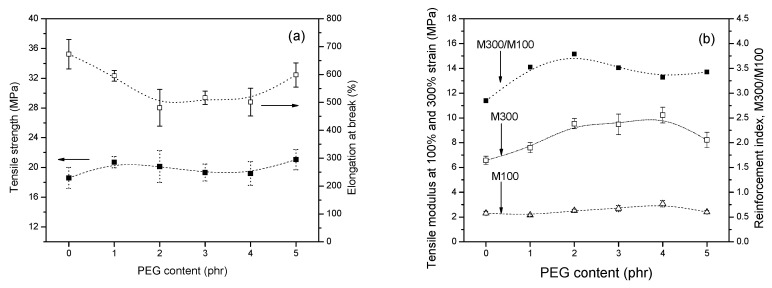
Effect of PEG contents on (**a**) tensile strength and elongation at break and (**b**) 100%, 300% modulus and reinforcement index (M300/M100) of silica/silane compounds.

The static mechanical behavior of the composites was analyzed with a tensile tester. When compared to the PEG 0, there were only small changes in the tensile strength with the increase of the PEG content, as displayed in Figure 9a, but a large rise in modulus at 300% extension in the range of 1–4 phr PEG could be found (Figure 9b). The results indicate that PEG can increase the stiffness of the silica-filled composites, while, at the same time, significantly improve the silica dispersion. The reinforcement index (RI), the ratio of the modulus at 300% elongation to that at 100% elongation, is related to the crosslink density and the strength of polymer–filler interactions. As the contribution of the filler network to the moduli was most pronounced at low elongations, the ratios of moduli at high and low elongations also gave an indication of the quality of silica dispersion. Such results can be explained by the fact that an appropriate content of PEG can improve the filler dispersion and increase the silanization efficiency, which results in the interfacial interaction between silica and matrix contributing to the stiffness of the materials. The use of excess PEG, which was uncombined with silica in the compound, i.e., 5 phr, resulted in the reduction of the modulus at high extension.

**Figure 10:**
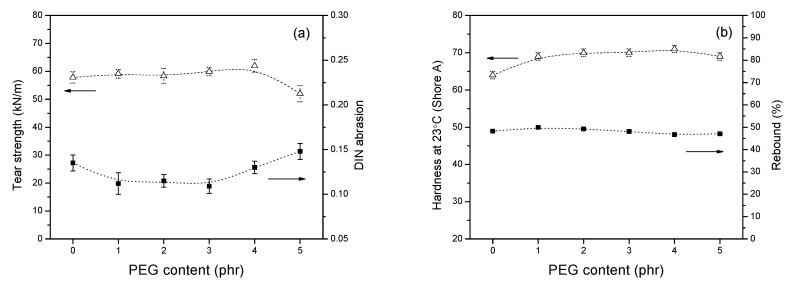
Effect of PEG contents on (**a**) tear strength and DIN abrasion and (**b**) hardness and rebound of silica/silane compounds.

The use of PEG further shows a slight improvement in tear strength and DIN abrasion properties, compared to the PEG-free system, as shown in Figure 10a. By increasing the content of PEG, tear strength of the vulcanizates initially rose to reach the maximum at 4 phr of PEG then decreased at the level lower than the reference PEG 0 composite. The changes of tear strength with the PEG content, therefore, showed a similar tendency and corresponded to the values of the tensile modulus at 300% elongation (M300), demonstrating that an appropriate concentration of PEG does enhance the strength of the rubber vulcanizates. As expected, the addition of PEG from 1 to 4 phr into the silica/silane compounds improved the abrasion resistance, indicated by the decrease of DIN abrasion values. This is owing to the improvement of the silica dispersion (Payne effect), which imparts improved wear resistance to the vulcanizates. In general, it has been reported that rubber vulcanizates with higher modulus show better wear resistance [37]. This increase in strength and abrasion will benefit tire tread performance.

The rebound resilience is used to characterize the energy recovery capability of rubber composite after elastic deformation, mostly caused by compressive stress. Generally, the rubber resilience is related to the flexibility of polymer chain, intermolecular force, crosslink density, filler dispersion and softener effect. As shown in Figure 10b, the increasing PEG content has only a minor influence on the property of rebound at room temperature. In contrast, the composites with different PEG content show higher hardness, comparing with the PEG 0 composite. The change in hardness corresponds to the crosslink density when the amount of PEG is increased, which shows a similar trend with the delta torque values (MH–ML).

**Figure 11:**
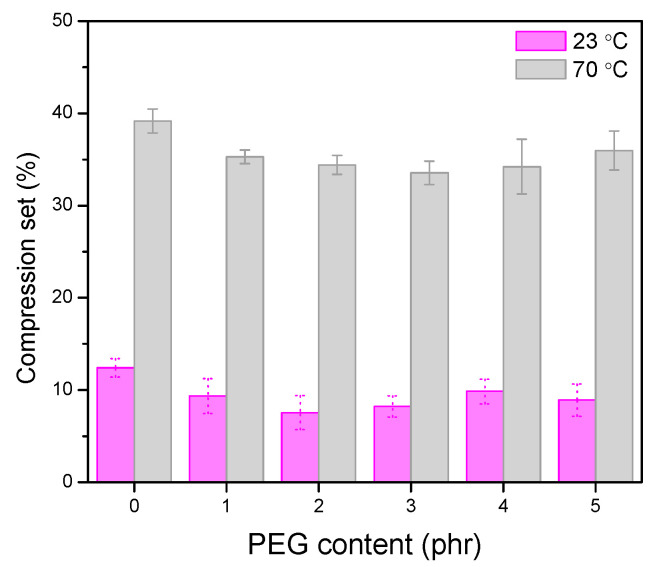
Effect of PEG contents on compression set at 23 °C and 70 °C.

The compression set of vulcanizates is one of the important properties to evaluate the static permanent deformation of rubber products. It is mainly dependent on the recovery ability of the polymer chains. Figure 11 illustrates the effect of PEG on the compression set of silica/TESPD vulcanizates at room temperature as well as at 70 °C with the testing time of 48 h. It can be seen that, with the addition of PEG, the compression set improved at both 23 °C and 70 °C, compared to the PEG 0 composite. With the increase of PEG content, the compression set decreased before a slight increase when the PEG content exceeded 3 phr. Normally, the recovery ability of the rubber chain not only depends on the rubber–rubber interaction, the rubber–filler interaction and the networks but also on the viscoelasticity of the polymers. On the one hand, the addition of PEG is beneficial to enhance the networks between silica and the matrix, and then the irreversible deformation can be reduced when the external forces were applied. On the other hand, as polymers display both elastic and viscous properties, PEG could increase the viscosity of the rubber compound, resulting in an increase of the permanent deformation. The influence of PEG on the compression set showed a similar trend with the performance of elongation at break and Mooney viscosity.

### 3.7. Dynamic Viscoelastic Properties

The response of viscoelastic performances to cyclic or sinusoidal deformations can be investigated by DMA–temperature sweeps. The storage modulus (E’) corresponds to the elastic deformation of rubber, and the loss modulus (E’’) corresponds to the viscous deformation. The mechanical loss factor is expressed as $tan\;\delta =E^{\prime \prime }/E\prime$, which indicates the occurrence of molecular mobility and segmental transitions, such as the glass transition temperature (T_g_).

**Figure 12:**
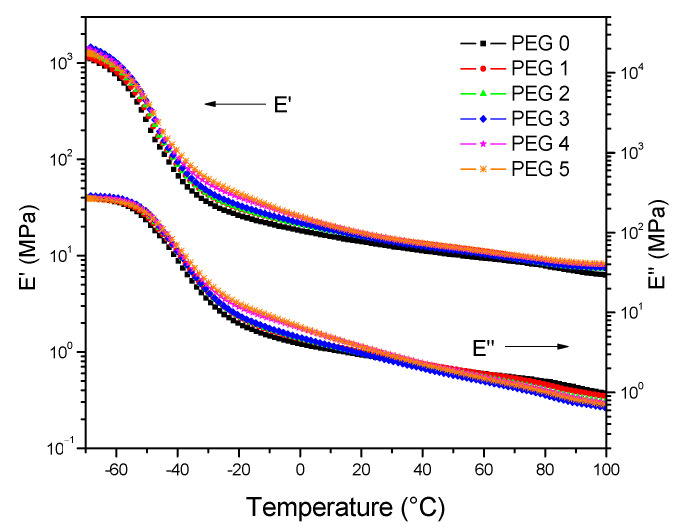
Dependences of the storage moduli E’ and the loss moduli E’’ on temperature.

In Figure 12, the temperature dependencies of the storage (E’) and loss (E’’) moduli of the indicated vulcanizates are given. Over the temperature range from −70 °C to 100 °C, the E’ of the PEG modified vulcanizates was higher than that of the vulcanizate without PEG (PEG 0). This finding can be attributed to the increase of reinforcement by filler [38]. The loss modulus (E”) of the PEG modified vulcanizates was higher than that of the vulcanizate without PEG (PEG 0) in the low temperature range (appr. <40 °C), which shows an opposite tendency, comparing with the E” in the high temperature range (approximately >40 °C). As the E’’ is mainly caused by the friction between filler particles, this finding demonstrates that PEG influences the polymer–filler interaction.

**Figure 13:**
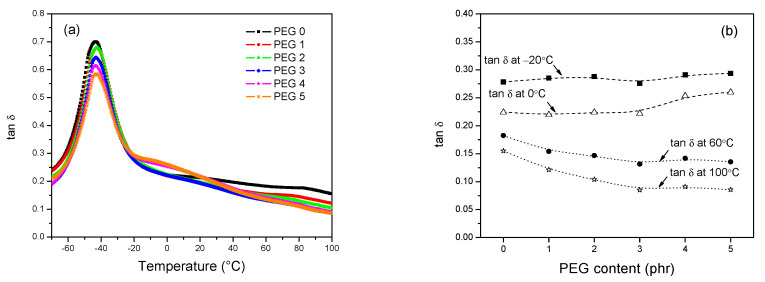
(**a**) Temperature dependence of tan δ for the indicated vulcanizates and (**b**) the values of tan δ at different temperatures.

The dependencies of tan δ on temperature are given in Figure 13a. With the addition of PEG, the dynamic glass transition temperature (Tg) of the composites was found to shift to higher temperatures, as compared to that of the composite without PEG (PEG 0), which is due to the increase of the crosslink density. The results were supported by static glass transition temperature of the composites performed by differential scanning calorimetry (DSC) in supporting information (Figure S2). Furthermore, in laboratorial analysis, DMA is widely utilized to predict tire tread performance with regard to wet traction and rolling resistance; tan δ at 0 °C is close to the temperature range, in which an increased value indicates an improvement of wet skid resistance of a tire, while the tan δ in the temperature range >40 °C, in particular tan δ at 60 °C, of vulcanizates indicates the loss of energy under dynamic deformation, in which the reduced tan δ is a clear indication for the reduction of the rolling resistance of a tire tread [39,40]. It is known that the rubber compound with high resilience has higher friction on ice in the temperature range of −20 °C, and, for this reason, excellent snow grip properties of vulcanizates can be predicted by lower values of tan δ at −20 °C [41,42]. In contrast, the rubber compound with low loss of energy at 100 °C indicates low heat buildup. Figure 13b shows the tan δ at −20 °C, 0 °C, 60 °C and 100 °C of the silica/silane compounds at various PEG content from DMA measurements. The increase of PEG content in the silica/silane composites increased the tan δ at −20 °C and 0 °C, continuously. This behavior is probably due to the Tg of pure PEG, which generates a tan δ peak at the temperature range of −25 to 0 °C. As a consequence, with regard to ice traction and wet grip, the increasing content of PEG gave positive effects on both properties. The tan δ values at 60 °C and 100 °C showed the same trend that they significantly decreased when a small amount of PEG was added (i.e., 1–3 phr); thereafter, a slightly reduction was observed. Therefore, rolling resistance and heat buildup of the vulcanizate could also be improved with the employment of PEG in the system. It is concluded that PEG reduces the filler fraction in the compound.

To evaluate the possibility of practical application of PEG in green tire tread, the effects of PEG on the magic triangle properties were compared with the typical tread formulation. When 3 phr of PEG was introduced into the silica/silane composite (PEG 3), all the three magic triangle properties, including rolling resistance and abrasion resistance, improved 39% and 24%, respectively, compared with the composite without PEG (PEG 0). The addition of 4 phr PEG in the compound did reduce the rolling resistance by 29% and increase the wet grip by 13%, respectively, suggesting that the excellent fuel consumption and driving safety are affected. As observed also for other properties earlier, the use of PEG, for example 3–4 phr, as an interfacial modifier in the silane-modified silica composites showed optimum properties, which indicates the best balance of both physical and chemical interactions and provides a solution for solving “magic triangle” in the green tires for passenger cars.

## 4. Conclusions

The addition of a small amount of PEG 4000 as an interfacial modifier on the second mixing stage in silane-modified silica composites improved the properties of Mooney viscosity; cure rate index; tensile strength; tensile modulus at 300% elongation; tear strength; DIN abrasion resistance; compression set at both 23 and 70 °C, due to an increase in the crosslink density; and silica dispersion. The dynamic mechanical properties, including rolling resistance, wet skid performance, ice traction and heat buildup were also improved, simultaneously. Summarizing from the perspective of tire performance, at optimum PEG content of 3–4 phr, the “magic triangle” properties were improved simultaneously, compared to the silica/silane composites. PEG can shield the silanol groups on the silica surface, leading to shortened vulcanization time, which enables an increase in productivity and energy saving. However, the excess PEG acted as lubricant and increased the possibility to shield the disulfide bonds of TESPD and resulted in the reduction of crosslink density and physical mechanical properties. An optimum PEG content is necessary for the overall performance and polymer–filler interaction. The results also demonstrated that the interfacial modifier, applied in green tire technology, physiosorbed/chemisorbed on the silica surface would improve of the performance, especially for the dynamic mechanical properties of tire tread. This finding will improve our understanding of the “magic triangle” performance of silica-loaded tire tread vulcanizates in the future.

## Figures and Tables

**Table 1:** Formulation for the modified silica.

Materials	PEG 0-S	PEG 1-S	PEG 2-S	PEG 3-S	PEG 4-S	PEG 5-S
Silica (g)	80	80	80	80	80	80
TESPD (g)	6.4	6.4	6.4	6.4	6.4	6.4
PEG 400 (g)	0	1	2	3	4	5

**Table 2:** Formulation for the composites in phr (parts in weight per 100g rubber).

Materials	PEG 0	PEG 1	PEG 2	PEG 3	PEG 4	PEG 5
SBR	80	80	80	80	80	80
BR	20	20	20	20	20	20
Silica	80	80	80	80	80	80
TESPD	6.4	6.4	6.4	6.4	6.4	6.4
Naphthenic oil	10	10	10	10	10	10
PEG	0	1	2	3	4	5
Stearic acid	2	2	2	2	2	2
Zinc oxide	3	3	3	3	3	3
Vulkanox^®^ 4020	2	2	2	2	2	2
Antilux^®^ 500	1	1	1	1	1	1
CBS	1.6	1.6	1.6	1.6	1.6	1.6
DPG	1.5	1.5	1.5	1.5	1.5	1.5
Sulfur	2.0	2.0	2.0	2.0	2.0	2.0

**Table 3:** Weight losses of modified silica.

Weight Loss/%	PEG 0-S	PEG 1-S	PEG 2-S	PEG 3-S	PEG 4-S	PEG 5-S
30–110 °C	1.73	1.76	1.85	1.90	2.00	2.05
110–800 °C	4.03	4.07	4.11	4.39	4.94	4.91

**Table 4:** Relative intensity of the peak at 3350 cm^−1^ and 1630 cm^−1^ for modified silica.

RI	PEG 0-S	PEG 1-S	PEG 2-S	PEG 3-S	PEG 4-S	PEG 5-S
3350 cm^−1^	0.152	0.118	0.101	0.080	0.074	0.087
1630 cm^−1^	0.787	0.028	0.029	0.029	0.023	0.025

## Data Availability

The data presented in this study are available on request from the corresponding author.

## Supplementary Materials

The following are available online at https://www.mdpi.com/2073-4360/13/5/788/s1, Figure S1: TGA curves of the modified silica, Figure S2: Differential scanning calorimetry curves and Tg for the indicated composites.
