# Supplementary material for: Synergistic Effect by Polyethylene Glycol as Interfacial Modifier in Silane-Modified Silica-Reinforced Composites
Source: Polymers (Basel). 2021 Mar 4;13(5):788. doi: 10.3390/polym13050788 (PMC7961832; doi:10.3390/polym13050788)
Supplement: Supplementary file 1 [file polymers-13-00788-s001.pdf]

Supporting information:

Synergistic Effect by Polyethylene Glycol as Interfacial Modifier in Silane Modified Silica Reinforced Composites

Minghan Xu, Hao Xue, Wit Yee Tin, He Wang, Zhanfu Yong and Qingfu Wang*

Key Laboratory of Rubber-Plastics, Ministry of Education/Shandong Provincial Key Laboratory of Rubber-plastics, School of Polymer Science and Engineering, Qingdao University of Science & Technology, Qingdao 266042, China.

* Corresponding Author

Email: wangqf@qust.edu.cn;

Tel.: +86-532-8402-2768

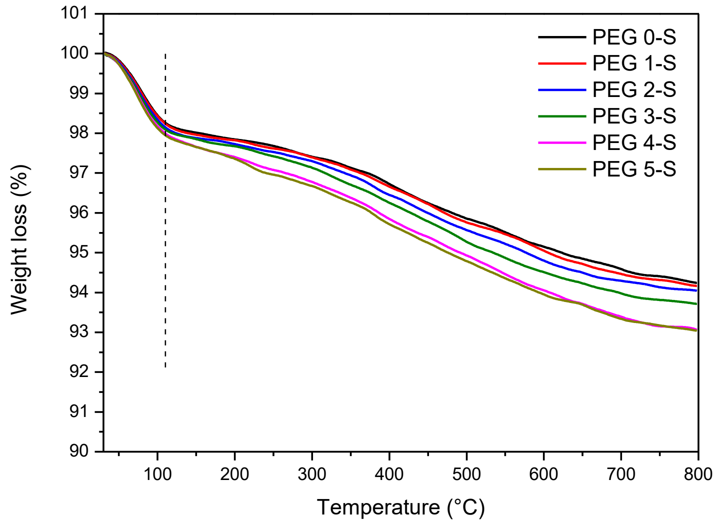

Figure S1. TGA curves of the modified silica.

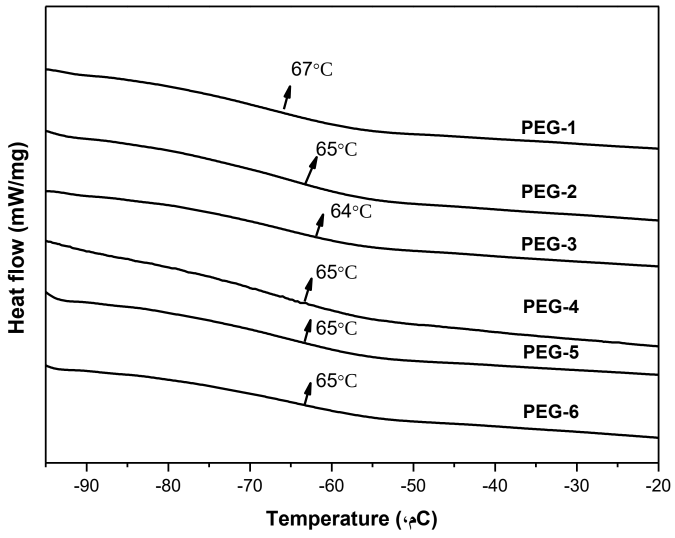

Figure S2. Differential scanning calorimetry curves and Tg for the indicated composites.
